# Xpert MTB/XDR performance in non-sputum samples for diagnosis of drug-resistant tuberculosis at a North Indian tertiary care center

**DOI:** 10.1128/jcm.01401-25

**Published:** 2026-06-12

**Authors:** Binit Kumar Singh, Pankaj Jorwal, Nishant Thakur, Manish Soneja, Naveet Wig, Ankita Anand, Shikha Dhawan

**Affiliations:** 1 Department of Medicine, All India Institute of Medical Sciences 28730 , New Delhi, India; 2 Centre for Social Integration and Borderless World 727370 , Uttar Pradesh, India; University of Western Australia, Perth, Western Australia, Australia

**Keywords:** Xpert MTB/XDR assay, Xpert MTB/ RIF ultra, line probe assay, liquid culture & DST

## Abstract

Rapid diagnosis of drug-resistant tuberculosis (DR-TB) is very challenging, specifically in extra-pulmonary TB specimens. The current study was performed on non-sputum specimens. All specimens were subjected on Xpert MTB/RIF Ultra followed by Xpert MTB/XDR, Ziehl-Neelsen (ZN) Staining, liquid culture, line probe assay (LPA), and drug susceptibility testing (DST). Out of 1,152 non-sputum specimens, 218 were observed to be positive on Xpert MTB/RIF Ultra and were selected for further investigation. From 218 MTBC positive, Xpert MTB/XDR yielded 174 (79.8%) positive while 25 (11.5%) were negative. On comparison with reference standards, ZN smear, and culture, the sensitivity was 100% and 95.51% with specificity of 17.86% and 18.63%, respectively. DST sensitivity and specificity were 84.62% and 96.61%, 83.33% and 96.88%, 50% and 98.75%, 50% and 98.72%, and 50% and 98.75%, respectively, for isoniazid, fluoroquinolones, amikacin, kanamycin, and capreomycin in culture positive specimens. The sensitivity and specificity of Xpert MTB/XDR assay, compared with LPA (MTBDR*plus* and MTBDR*sl*), was between 94.12%–100% and 98.39%–100%, respectively. Sensitivity and specificity of Xpert MTB/XDR matched with culture-DST and LPA results for all the tested drugs. Its placement in diagnostic algorithms with Xpert MTB/RIF Ultra under National TB Programs will enhance patient outcome by providing prompt switch to a DST guided treatment regimen.

**IMPORTANCE:**

This study provides crucial evidence supporting the use of Xpert MTB/XDR for rapid detection of DR-TB in non-sputum specimens including extra-pulmonary specimens, where diagnosis remains challenging due to paucibacillary in nature. By demonstrating high sensitivity and specificity compared with culture-DST and LPA, the assay shows strong potential to be integrated into the National TB Elimination Program (NTEP) diagnostic algorithm. Early detection of resistance to isoniazid, fluoroquinolones, and second-line injectables enables timely transition to DST-guided treatment, reducing delays and improving patient outcomes. The findings highlight the assay’s ability to reduce dependency on conventional laboratory-based DST, shortening turnaround time and strengthening decentralized diagnostic capacity. This study provides programmatic evidence for policy-level adoption, supporting India’s efforts toward DR-TB control and elimination.

## INTRODUCTION

Tuberculosis (TB) is a severe global health problem, caused by the bacteria *Mycobacterium tuberculosis* (MTB). The emergence of multiple drug-resistant (MDR) forms of MTB particularly extensively drug-resistant TB (XDR TB) has become a significant public health challenge. XDR-TB is defined as TB that is resistant to at least four of the potent anti-TB drugs: isoniazid (INH), rifampicin (RIF), which are the first line (FL) anti-TB drugs, as well as fluoroquinolone (FLQ), and bedaquiline/ linezolid (1). This level of resistance severely limits treatment options and is associated with higher morbidity and mortality rates.

According to the World Health Organization (WHO), there were an estimated 10.8 million new TB cases in 2023, of which approximately 400,000 were MDR-TB (1, 2), and around 6% of these MDR-TB cases were classified as XDR-TB. The incidence of XDR-TB varies significantly by region, with higher rates reported in countries such as India, China, and Russia. The treatment success rate for XDR-TB is alarmingly low, often less than 40%, highlighting the critical need for early and accurate detection (1).

Diagnosis and treatment of DR-TB is challenging in middle- and low-income countries due to multifold constraints of logistics, expertise, and resource availability. Additionally, if the site of infection is Extra Pulmonary (EP), then the situation becomes even more challenging; because of the paucibacillary nature of the disease and absence of a suitable sample site or type, all these factors make the management a difficult journey often traversed by patients, who starts at primary level but eventually ends up at a tertiary center before a concrete treatment plan is made and executed. Advanced imaging techniques and molecular diagnostics are required in such scenarios. The growing number of EPTB, especially at higher centers, highlights the critical need for more precise diagnostic techniques to detect MTB in EP cases (3). Traditional diagnostic methods, which were mainly created for pulmonary tuberculosis (PTB), frequently fail to identify EP cases due to its paucibacillary nature, leading to major treatment delays and worse outcomes for patients. New developments in molecular diagnostics, such as the TB-LAMP assay (4), Truenat assay (Molbio Diagnostics, India), and PCR-based methods, can potentially improve the sensitivity and specificity for the detection of DR MTB testing. Line Probe Assay (LPA) (MTBDR*plus* and MTBDR*sl*, Hain Life Sciences, Germany) recommended by WHO for both first-line (FL) and second-line (SL) molecular drug susceptibility testing (DST) showed excellent results specifically in PTB cases (5). Yet, the use of LPA in EPTB specimens showed mixed results, being useful in pus specimens. In other EPTB specimens, LPA did not represent its effectiveness as compared to Xpert MTB/RIF & MTB/RIF Ultra (Cepheid Inc., Sunnyvale, CA, USA) (6).

Recently, Cepheid Inc., Sunnyvale, CA, USA, introduced Xpert MTB/XDR cartridges and an advanced version of 10-colored Xpert instrument to detect genetic mutations associated with MTB and it targets INH (mutations at *katG, inhA* promoter, and *oxyR-aphC* intergenic region), ETH (mutations at the *inhA* promoter), FLQ (mutations at *gyrA* and *gyrB*), and SL Injectables (mutations at *rrs* and *eis* promoter) (7–9). The test kit facilitates comprehensive drug resistance profiling and is an improved diagnostic method as compared to Xpert MTB/RIF assay, LPA and Liquid Culture & DST (MGIT-960, Becton Dickinson, USA) (Reference). As Xpert MTB/XDR offers faster results (within 90 min) and does not require specialized laboratory settings, it is more accessible and quicker for field use in decentralized settings, improving timely treatment decisions. The best part being a comprehensive and detailed resistance profile in a single test to guide tailored treatment options. To the best of our knowledge, the use of Xpert MTB/XDR cartridge for testing specimens other than sputum or decontaminated sputum sediments has not been evaluated and reviewed by any TB controlling regulatory body. Thus, Xpert MTB/XDR can be a game changer for effective DST-driven management of drug-sensitive and drug-resistant TB in various specimen types, especially in EP sites, such as lymph nodes, biopsy, gastric aspirate, cerebral spinal fluid, and pleural fluid, which have very low yield on traditional culture-based methods (10–12).

The main objective of this study was to evaluate the efficiency and diagnostic accuracy of the Xpert MTB/XDR assay on non-sputum specimens. This study intended to explore the applicability of the Xpert MTB/XDR assay beyond traditional sputum samples, potentially enhancing diagnostic capabilities in diverse clinical specimens.

## RESULTS

**Fig 1:**
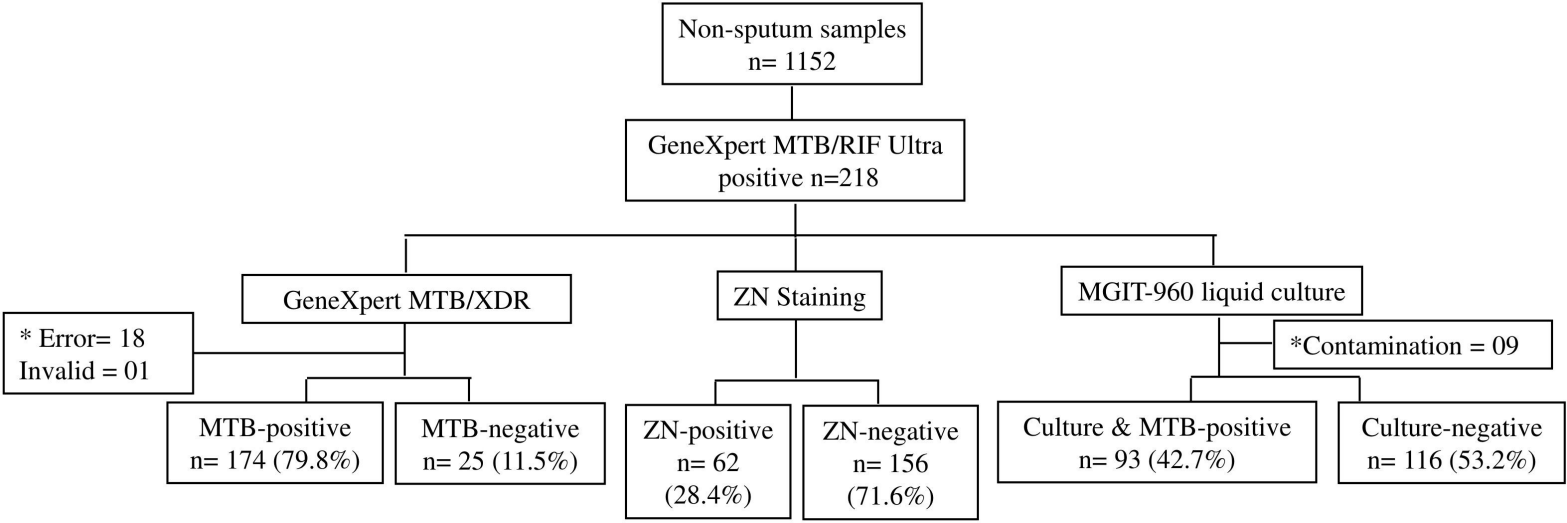
Workflow. Abbreviation: MTBC: *Mycobacterium tuberculosis* complexes; MGIT: Mycobacterium growth inhibitor tube; RIF: Rifampicin; INH: Isoniazid; ZN: Ziehl-Neelsen. *Error, invalid and contamination results were excluded from all comparisons.

Out of 1,152 EPTB samples tested, 218 (18.9%) were found to be Xpert MTB/RIF Ultra positive and were included for the study. The samples were evaluated using MGIT-960 liquid culture, and ZN smear staining. Among Xpert MTB/RIF Ultra (*n* =218) diagnosed MTB positive specimens, 93 (42.7%) and 62 (28.4%) samples were found positive using MGIT-960 liquid culture and ZN staining, respectively. While with Xpert MTB/XDR assay, 174 (79.8%) samples were positive, and 25 (11.5%) samples were negative for MTB (Fig. 1).

Among all Xpert MTB/RIF Ultra positives, comprising participants of all age groups, 20 (9%), 186 (85%), and 12 (6%) patients were of age groups 1–14 years, 15–60 years, and 61–84 years, respectively. Twenty-nine (13.3%) specimens were found to be RIF resistance, while 189 (86.7%) were RIF sensitive (Table 1).

**TABLE 1:** Demographics and sample type of patients^*a*^

S. no.	Category		Total no. *n*, %	Xpert Ultra cartridge *n*, %		Xpert MTB/XDR cartridge *n*, %			MGIT culture *n*, %		
				RIF-R	RIF-S	Positive	Negative	Error/INV	Positive	Negative	Contamination
1	Gender	Female	92, 42%	10, 34.5%	82, 43.4%	70, 40.2%	14, 56.0%	7/1,	36, 38.7%	52, 44.8%	04, 44.4%
		Male	126, 58%	19, 65.5%	107, 56.6%	104, 59.8%	11, 44.0%	11/0	57, 61.3%	64, 55.2%	05, 55.6%
2	Age	0–14 years	20, 09%	05, 17.2%	15, 7.9%	17, 9.8%	02, 8.0%	0/1	07, 7.5%	13, 11.2%	0
		15–60 years	186, 85%	24, 82.8%	162, 85.7%	148, 85.1%	21, 84.0%	17/0	80, 86.0%	97, 83.6%	09, 100%
		61–84 years	12, 6%	0	12, 6.3%	09, 5.2%	02, 8.0%	1/0	06, 6.5%	06, 5.2%	0
3	Sample types	BAL	85, 39%	13, 44.8%	72, 38.1%	70, 40.2%	09, 36%	6/0	55, 59.1%	27, 23.3%	03, 33.3%
		Biopsy/tissue	05, 2.3%	01, 3.4%	04, 2.1%	04, 2.3%	01, 4%	0/0	02, 2.1%	03, 2.6%	0
		Cold abscess	05, 2.3%	02, 6.9%	03, 1.6%	05, 2.9%	0	0/0	04, 4.3%	01, 0.9%	0
		CSF	02, 0.9%	0	02, 1.1%	01, 0.6%	0	1/0	0	02, 1.7%	0
		TBNA	07, 3.2%	01, 3.4%	06, 3.2%	05, 2.9%	02, 8%	0/0	01, 1.1%	06, 5.2%	0
		ETA	02, 0.9%	0	02, 1.1%	02, 1.1%	0	0/0	01, 1.1%	01, 0.9%	0
		GA	12, 5.5%	01, 3.4%	11, 5.8%	09, 5.2%	01, 4%	1/1	05, 5.4%	07, 6.0%	0
		Liver abscess	01, 0.5%	0	01, 0.5%	01, 0.6%	0	0/0	0	01, 0.9%	0
		LN	51, 23.4%	05, 17.2%	46, 24.3%	35, 20.1%	09, 36%	7/0	13, 14.0%	33, 28.4%	05, 55.6%
		PF	10, 4.6%	01, 3.4%	09, 4.8%	07, 4.0%	01, 4%	2/0	03, 3.2%	07, 6.0%	0
		Psoas abscess	01, 0.5%	0	01, 05%	01, 0.6%	0	0/0	0	01, 0.9%	0
		Pus	33, 15.1%	05, 17.2%	28, 14.8%	31, 17.8%	02, 8%	0/0	08, 8.6%	25, 21.6%	0
		Spinal aspirate	01, 0.5%	0	01, 0.5%	01, 0.6%	0	0/0	01, 1.1%	0	0
		Synovial fluid	02, 0.9%	0	02, 1.1%	01, 0.6%	0	1/0	0	01, 0.9%	01, 11.1%
		Urine	01, 0.5%	0	01, 0.5%	01, 0.6%	0	0/0	0	01, 0.9%	01, 11.1%
		Total	218, 100%	29, 13.3%	189, 86.7%	174, 79.8%	25, 11.5%	18/1, 8.7%	93, 42.7%	116, 53.2%	9, 4.1%

^*a*^ MTB: *Mycobacterium tuberculosis*; BAL; bronchoalveolar lavage: CSF: cerebrospinal fluid; TBNA; transbronchial needle aspiration; ETA: endotracheal aspirate; GA: gastric aspirate; LN: lymph node; PF: pleural fluid.

On comparison between Xpert MTB/RIF Ultra positives with Xpert MTB/XDR, 152 (69.7%) samples were found to be MTB positive with complete interpretable (with RIF resistance status) results and 22 (10.1%) samples were MTB positive with incomplete (without RIF resistance status) results. Rest 25 (11.5%) were observed MTB negative while error and invalids were found in 18 (8.3%) and one (0.4%) specimen, respectively (see Table S1 at https://doi.org/10.5281/zenodo.19913747).

Analysis of total MTB positivity drug resistance status and indeterminate results in Xpert MTB/XDR showed 55 (31.6%) samples to be INH resistant including four with low level resistance and 2.3% indeterminates. Moreover, 8%, 20.7%, 1.7%, 4%, and 1.7% samples were resistant to ETH, FLQ, AMK, KAP, and CAP, respectively (Table 2).

**TABLE 2:** Indeterminate and resistance detected with Xpert MTB/XDR in non-sputum specimens^*a*^

Name of drugs	Indeterminate MTB positive (*n*/*N*, %)	Resistance (*n*/*N*, %)	Sensitive (*n*/*N*, %)
INH	04/174, 2.3	55/174, 31.6 (four low level)	115/174, 66.1
ETH	02/174, 1.1	14/174, 08	136/174, 78.1
FLQ	11/174, 6.3^*b*^	36/174, 20.7 (three low level)	140/174, 80.5
AMK	11/174, 6.3	03/174, 1.7	156/174, 89.7
KAN	11/174, 6.3^*c*^	07/174, 04	159/174, 91.4
CAP	12/174, 6.9	03/174, 1.7	158/174, 89.7

^*a*^ AMK: amikacin; CAP: capreomycin; ETH: ethionamide; FLQ: fluoroquinolones; INH: isoniazid; KAN: kanamycin; MTB: *Mycobacterium tuberculosis*; *N*: number.

^*b*^ One sample was found to be missing of *gyrA1, gyrA2 *&* gyrA3* genes.

^*c*^ In one sample mutation at *eis *mutB was observed.

Comparison with ZN-smear microscopy demonstrated a sensitivity of 100% (CI: 93.94–100), indicating the detection of true positive cases, but with low specificity 17.86% (CI: 11.9–25.22), reflecting a high rate of false positives. This is corroborated by the PPV of 33.91% (CI: 32.2–35.66) and NPV of 100% (CI: 86.28–100). In contrast, the MGIT culture method showed slightly lower sensitivity at 95.51% (CI: 88.89–98.76) but similar low specificity at 18.63% (CI: 11.6–27.55) with ZN smear. The PPV was higher for MGIT culture at 50.60% (CI: 48.02–53.17), while the NPV was representable at 82.61% (CI: 62.67–93.07) (Table 3).

**TABLE 3:** Comparison of Xpert MTB/XDR with ZN-smear and MGIT culture for the detection of MTB in non-sputum specimens^*a*^

	TP	FN	FP	TN	Sensitivity (CI)	Specificity (CI)	PPV (CI)	NPV (CI)
ZN smear^*b*^	59	0	115	25	100 (93.94–100)	17.86 (11.9–25.22)	33.91 (32.2–35.66)	100 (86.28–100)
MGIT culture^*c*^	85	4	83	19	95.51 (88.89–98.76)	18.63 (11.6–27.55)	50.60 (48.02–53.17)	82.61 (62.67–93.07)

^*a*^ CI: confidence interval; FN: false negatives; FP: false positives; TN: true negatives; TP: true positives.

^*b*^ Invalid and errors on Xpert and were excluded.

^*c*^ Invalid, error, and contamination in MGIT culture were excluded.

Among MGIT culture positive specimens (*n* = 93, 42.7%), only valid and interpretable results associated with particular drugs were taken for the final analysis of MGIT-DST. For INH (*n*= 85), the Xpert MTB/XDR assay showed high sensitivity (84.62%) and specificity (96.61%), indicating reliable performance in detecting true positives and true negatives. The assay also demonstrated high sensitivity and specificity FLQ—83.33% (CI: 58.58–96.42) and 96.88% (CI: 89.16–99.62), respectively. However, for AMK, KAN, and CAP, the sensitivity dropped to 50% although specificity remains high (98.72%–98.75%). We could not perform DST of the ETH drug due to non-availability of drug during the study period (Table 4).

**TABLE 4:** Comparison of Xpert MTB/XDR with MGIT culture & DST in non-sputum specimens^*a*^

Drugs	*N*	TP	FN	FP	TN	Sensitivity (CI)	Specificity (CI)	PPV (CI)	NPV (CI)
INH	85	22	04	02	57	84.62 (65.13–95.64)	96.61 (88.29–99.59)	91.67 (73.61–97.75)	93.44 (85.25–97.23)
FLQ	82	15	03	02	62	83.33 (58.58–96.42)	96.88 (89.16–99.62)	88.24 (65.37–96.75)	95.38 (88.02–98.31)
AMK	82	01	01	01	79	50.00 (1.26–98.74)	98.75 (93.23–99.97)	50.00 (8.39–91.61)	98.75 (95.18–99.68)
KAN	82	02	02	01	77	50.00 (6.76–93.24)	98.72 (93.06–99.97)	66.67 (18.44–94.65)	97.47 (93.53–99.03S)
CAP	82	01	01	01	79	50.00 (1.26–98.74)	98.75 (93.23–99.97)	50.00 (8.39–91.61)	98.75 (95.18–99.68)
ETH^*b*^	NA	NA	NA	NA	NA	NA	NA	NA	NA

^*a*^ DST: drug susceptibility testing; *N*: Number of DST performed on culture positive specimens; AMK: amikacin; CAP: capreomycin; ETH: ethionamide; FLQ: fluoroquinolones; INH: isoniazid; KAN: kanamycin; MTB: *Mycobacterium tuberculosis*; *N*: number; CI: confidence interval; FN: false negatives; FP: false positives; TN: true negatives; TP: true positives; NA: not applicable.

^*b*^ DST of ETH was not performed due to unavailability of the drug during the study period. Three specimens were found to be indeterminate on Xpert MTB/XDR for FLQ, AMK, KAN, & CAP.

While analyzing the frequencies of single mutation in drug specific genes, for INH, mutations were found predominantly at *katG* gene (76.4%) and the *inhA* promoter (27.3%), with lower frequencies in the *fabG1* (3.6%) and the *oxyR-ahp C* intergenic region (10.9%). ETH showed a 100% mutation rate in the inhA promoter. Similarly, FLQ exhibited multiple mutations across both *gyrA* and *gyrB* genes, with *gyrA1 mutB* and *gyrA3 mutB* being the most common with the same frequency of 47.2% for each gene. For AMK and CAP, the *rrs* gene mutations were present in all samples (100%). KAN showed *rrs* gene mutations in 42.9% and *eis* promoter mutations in 57.1% specimens (see Table S2 at https://doi.org/10.5281/zenodo.19913747).

Whereas, after analyzing the frequency of multiple mutation patterns in single drugs, it was observed that in INH, the most common mutations were found in the *katG* gene (60%), followed by *inhA* (14.5%), with other mutations such as oxyR-ahpC and fabG1 were less frequent. Some specimens exhibited combined mutations in *katG* gene with *inhA* gene as the most frequent combination (9.1%). For FLQ, multiple combinations of mutations were observed across both *gyrA* and *gyrB* genes, with *gyrA3 mutB* being the most frequent (27.8%) (see Table S3 at https://doi.org/10.5281/zenodo.19913747).

Subsequently, we also analyzed the mutations in specifics genes with DR patterns and observed nine different types of combinations. Resistance in INH with the combination of FLQ drugs was found maximum with 9.2% (*n* = 16), like with INH mono resistance (*n* = 16, 9.2%) among all Xpert MTB/XDR positive specimens. Among these 16 specimens, 10 were RIF sensitive and 6 were RIF resistant. Consequently, FLQ mono resistance was observed in 11 (6.3%) specimens, which were all RIF sensitive in Xpert Ultra (Table 5).

**TABLE 5:** Frequencies of combined mutation patterns in multiple drug specific genes in complete results with MTB (*N* = 174)^*a*^

Drug combinations	RIF-S	RIF-R	INH associated mutations	FLQ associated mutations	SLID associated mutations	ETH associated mutations	No. of samples, %
INH (*n* = 16, 9.2%) (RIF-S= 10; RIF-R= 06)	08	05	*katG* mut				13, 81.3%
	01	0	*katG* mut + *oxyR* ahpC mut				01, 6.2%
	0	01	*fabG1* mut				01, 6.2%
	01	0	*oxyR-ahpC* mut + *fabG1* mut				01, 6.2%
INH+FLQ (*n* = 16, 9.2%) (RIF-S= 10; RIF-R= 06)	01	0	*katG* mut	*gyrA1* mutC + *gyrA3* mutC			01, 6.2%
	0	01	*katG* mut	*gyrA1* mutB + *gyrA2* mutA+ *gyrA3* mutB			01, 6.2%
	02	0	*katG* mut	*gyrA1* mutB + *gyrA2* mutA			02, 12.5
	01	0	*katG* mut	*gyrA1* mutB + *gyrA3* mutC			01, 6.2%
	04	03	*katG* mut	*gyrA3* mutB			07, 43.8%
	01	0	*katG* mut	*gyrA3* mutB + *gyrB2* mut			01, 6.2%
	0	01	*katG* mut + *oxyR* ahpC mut	*gyrA1* mutB + *gyrA2* mutA+ *gyrA3* mutB			01, 6.2%
	01	0	*oxyR-ahpC* mut	*gyrA1* mutB + *gyrA3* mutC			01, 6.2%
	0	01	*katG* mut + *inhA* mut	*gyrA1* mutA + *gyrA2* mutA			01, 6.2%
INH+FLQ+KAN (*n* = 01, 0.6%) (RIF-R= 01)	0	01	*katG* mut	*gyrA1* mutB + *gyrA2* mutA+ *gyrA3* mutB	*eis* mutB		01, 100%
INH+FLQ+ETH (*n* = 02, 1.1%) (RIF-S= 01; RIF-R= 01)	01	0	*katG* mut + *inhA* mut	*gyrA1* mutB		*inhA* mut	01, 50%
	0	01	*katG* mut + *inhA* mut + *oxyR* ahpC mut	*gyrA1* mutA + *gyrA2* mutA + *gyrB2* mut		*inhA* mut	01, 50%
INH+ETH (05, 2.9%) (RIF-S= 05)	03	0	*inhA* mut			*inhA* mut	03, 60%
	02	0	*katG* mut + *inhA* mut			*inhA* mut	02, 40%
INH+FLQ+AMK+KAN+CAP (*n* = 01, 0.6%); (RIF-S= 01)	01	0	*katG* mut	*gyrA1* mutC	*rrs* mut		01, 100%
FLQ (*n* = 11, 6.3%) (RIF-S= 11)	02	0		*gyrA1* mutB			02, 18.2%
	01	0		*gyrA1* mutB + *gyrA2* mutA			01, 9.1%
	01	0		*gyrA1* mutB + *gyrA2* mutA+ *gyrA3* mutB			01, 9.1%
	01	0		*gyrA1* mutB + *gyrA3* mutC			01, 9.1%
	01	0		*gyrA1* mutB + *gyrA3* mutB			01, 9.1%
	02	0		*gyrA1* mutC			02, 18.2%
	02	0		*gyrA3* mutB			02, 18.2%
	02	0		*gyrB2* mut			01, 9.1%
FLQ+KAN (*n* = 01, 0.6%) (RIF-S= 01)	01	0		*gyrA1* mutB + *gyrA3* mutC	*eis* mutB		01, 100%
AMK+KAN+CAP (*n* = 01, 0.6%) (RIF-S= 01)	01	0			*rrs* mut		01, 100%

^*a*^ *n*: number of samples; SLID: second line injectable drugs (AMK, KAN, & CAP); AMK: amikacin; KAN: kanamycin; CAP: capreomycin; ETH: ethionamide; FLQ: fluoroquinolones; INH: isoniazid; RIF: rifampicin; R: resistant; S: sensitive; Only complete results with MTB and resistant to any drugs were included (*n *= 54), any indeterminates results in any drugs were excluded.

An important finding in this study was incomplete DST results on Xpert MTB/XDR assay that underscore the challenges in determining drug resistance patterns in DRTB. As per our observations, 2.3% of the samples showed incomplete resistance profiles for INH, where mutations in the *katG* gene were identified, but resistance to other drugs like FLQ or SL injectable drugs (SLID) was indeterminate. Additionally, 1.1% of samples exhibited dual resistance to INH and FLQ, with mutations detected in both *katG gene* and *gyrA* or *gyrB* genes, while resistance to SLID remained unclear. These incomplete results emphasize the need for supplementary testing methods to accurately determine drug resistance profiles in TB cases where the Xpert MTB/XDR assay does not provide comprehensive data. Addressing these gaps will be crucial for optimizing treatment strategies and improving outcomes for DRTB patients (see Table S4 at https://doi.org/10.5281/zenodo.19913747).

When we compared the performance of the Xpert MTB/XDR assay with the MTBDR*plus* and MTBDR*sl* assays, for INH resistance, the Xpert MTB/XDR assay showed a high sensitivity of 95.65% and a specificity of 98.39% compared to the MTBDRplus assay, indicating its effectiveness in accurately detecting INH resistance. Similarly, for FLQ, the Xpert MTB/XDR assay demonstrated a sensitivity of 94.12% and a perfect specificity of 100% when compared to the MTBDR*sl* assay. The assay also exhibited excellent performance for SLID, with 100% sensitivity and specificity, as observed with the MTBDR*sl* assay. These results highlight the robustness of the Xpert MTB/XDR assay in identifying drug resistance across different drug classes, supporting its reliability and utility in the comprehensive management of DRTB (Table 6).

**TABLE 6:** Comparison of Xpert MTB/XDR with MTBDR*plus* and MTBDR*sl* in non-sputum MGIT culture positive specimens^*a*^

Drugs	*N*	TP	FN	FP	TN	Sensitivity (CI)	Specificity (CI)	PPV (CI)	NPV (CI)
INH (MTBDR*plus*)	85	22	01	01	61	95.65 (78.05–99.89)	98.39 (91.34–99.96)	95.65 (75.86–99.35)	98.39 (89.97–99.76)
FLQ (MTBDR*sl*)	82	16	01	0	65	94.12 (71.31–99.85)	100 (94.48–100)	100 (79.41–100)	98.48 (90.66–99.77)
SLID (MTBDR*sl*)	82	2	0	0	80	100 (15.81–100)	100 (95.49–100)	100 (15.81–100)	100 (95.49–100)

^*a*^ DST: drug susceptibility testing; *N*: Number of line probe assay (MTBDR*plu*s and MTBDR*sl*) performed on culture positive specimens; SLID: second line injectable drugs (AMK, KAN, & CAP); AMK: amikacin; KAN: kanamycin; CAP: capreomycin; ETH: ethionamide; FLQ: fluoroquinolones; INH: isoniazid; MTB: *Mycobacterium tuberculosis*; *N*: number; CI: confidence interval; FN: false negatives; FP: false positives; TN: true negatives; TP: true positives.

## DISCUSSION

The current study is the initial one to evaluate Xpert MTB/XDR in non-sputum specimens. We selected 218 Xpert MTB/RIF Ultra positive non-sputum specimens from a total of 1,152 non-sputum specimens. While comparing the positivity of assay in different specimens, BAL showed maximum positivity with 40.2%, followed by lymph node (20.1%) and pus (17.8%). The findings of this study highlight the significant potential of the Xpert MTB/XDR assay in advancing the diagnosis and management of DRTB, particularly in non-sputum specimens. The study found high sensitivity and specificity in detecting MTB and drug resistance, aligning with recent studies that show the effectiveness of Xpert assays in diverse clinical settings. For instance, a study found that the Xpert MTB/RIF Ultra assay had a sensitivity of 95.2% and specificity of 98.9% in detecting MTB in both pulmonary and extrapulmonary specimens (13). In our study, the Xpert MTB/XDR assay showed a sensitivity of 100% for detecting MTB in non-sputum samples, corroborating these high sensitivity rates.

The study identified significant resistance patterns, with 31.6% of samples showing resistance to INH, 8% to ETH, 20.7% to FLQ and SLID. Many studies have demonstrated the utility of the Xpert MTB/XDR assay in detecting resistance to INH, FLQ, and SLIDs, highlighting its ability to identify critical drug resistance mutations (14–16). This comprehensive resistance profiling is crucial for guiding effective treatment strategies, especially in regions with high rates of MDR-TB and XDR-TB.

One of the key advantages of the Xpert MTB/XDR assay is its rapid turnaround time, providing results in less than 90 min. This rapid diagnostic capability is crucial for initiating timely treatment, as emphasized by a previous study, which noted the importance of quick and accurate results in TB management (17). The prompt availability of diagnostic information can significantly enhance patient outcomes by reducing delays in treatment initiation. Similar findings have been shown in other studies highlighting the effectiveness of the Xpert MTB/RIF Ultra assay in diagnosing TB and RIF resistance status from non-sputum samples, including pleural fluid and lymph node biopsies (18, 19). However, the current study deployed diverse clinical specimens and thus demonstrated expanded utility of the Xpert MTB/XDR assay in various healthcare settings with existing Xpert MTB/RIF Ultra assay.

Further supporting the relevance and accuracy of these findings, there have been many studies emphasizing advancements in molecular diagnostics that enhance the detection of MTB and drug-resistance mutations (8, 20). There have been many studies confirming the diagnostic accuracy of Xpert assays across different specimen types, highlighting the adaptability and precision in varied clinical scenarios (12, 21). Additionally, some reports discuss the importance of molecular diagnostics in improving TB detection rates among vulnerable populations such as those with HIV (22–24). Few studies illustrate the efficacy of Xpert assays in diagnosing TB from pleural samples and managing DR TB, respectively, reinforcing the study’s implications for clinical practice (25, 26).

Our study reveals that a notable proportion of samples exhibited indeterminate results for drug resistance, with incomplete resistance profiles as observed for INH, FLQ, and SLID. Specifically, 2.3% of samples showed incomplete results for INH, where mutations in the *katG* gene were present while resistance to other drugs remained unclear. Additionally, 1.1% of samples demonstrated dual resistance to INH and FLQ, with identifiable mutations in both *katG* and *gyrA* or *gyrB* genes, although resistance patterns for other drugs were not fully determined. These findings highlight the inherent limitations of the assay in cases where results are not entirely conclusive. The presence of partial resistance profiles underscores the need for additional diagnostic methods or confirmatory testing to achieve a comprehensive understanding of drug resistance. Addressing these limitations is critical for ensuring comprehensive management of DRTB cases (see Table S4 at https://doi.org/10.5281/zenodo.19913747).

The Xpert MTB/XDR assay shows diagnostic accuracy and comprehensive drug resistance profiling in non-sputum samples, addressing a critical gap in TB diagnostics. While analyzing the data from mutations and DST, all 11 (6.3%) FLQ mono-resistant were found to be RIF sensitive and results were comparable with other studies conducted in the same settings in India (27). This finding would be more challenging before starting the treatment under a national program. Its ability to provide rapid and detailed results can significantly enhance TB management, particularly for patients with EPTB, who are often challenging to diagnose with traditional methods. Future research should focus on validating these findings in larger and more diverse populations, as well as exploring the assay’s potential in various clinical settings to maximize its impact on global TB control efforts.

### Limitations

While the Xpert MTB/XDR assay demonstrates significant advantages, it is important to acknowledge its limitations and potential drawbacks: The Xpert MTB/XDR assay, while offering rapid results and comprehensive drug resistance profiling, can be a bit costly. This may limit its accessibility in resource-limited settings where the burden of TB and DRTB is high. Although the assay has high sensitivity, no diagnostic test is perfect. There is always a risk of false positives and false negatives. False positives can occur due to contamination or cross-reactivity, while false negatives might arise in cases with very low bacterial loads or due to mutations not detected by the assay. The assay may not detect all possible mutations associated with drug resistance. For example, while many common resistance mutations are covered, rare or novel mutations might not be identified, potentially leading to incomplete resistance profiling.

Although the assay can handle a variety of non-sputum specimens, performance may vary depending on the specimen type and quality. For instance, specimens with low bacterial load or high contamination rates might impact the accuracy of the test results. The need for specialized equipment and trained personnel to operate the Xpert MTB/XDR assay can be a barrier in certain settings. Moreover, maintaining and calibrating the equipment requires ongoing technical support and quality control. The study primarily focuses on the assay’s diagnostic performance. There is limited information on how the assay’s use influences long-term patient outcomes, including treatment success rates and overall impact on TB control programs.

### Conclusion

This study highlights the significant potential of the Xpert MTB/XDR assay in enhancing the diagnosis and management of drug-resistant tuberculosis (DR-TB), particularly in non-sputum specimens. With its representable sensitivity and specificity, the assay provides a rapid and comprehensive drug resistance profile, which is crucial for tailored treatment strategies. Its performance in detecting TB and DRTB for critical drugs, such as isoniazid, fluoroquinolones, and injectable drugs, positions it as a valuable diagnostics tool, especially in EPTB cases where traditional diagnostic methods often showed limitations. The rapid turnaround time of the assay, providing results in under 90 min, further contributes to the timely initiation of appropriate treatment, improving patient outcomes. Future studies are essential to validate these findings in larger, more varied populations and to assess the long-term impact of the assay on patient outcomes and TB control efforts.

## MATERIALS AND METHODS

### Study design

The study was designed as a cross-sectional evaluation of the Xpert MTB/XDR assay’s performance on non-sputum specimens. The study samples were collected and processed between October 2023 to June 2024.

### Sample collection

A total of 1,152 non-sputum samples were collected and processed. Major samples included were bronchoalveolar lavage (BAL, 39%), lymph node aspirate (LN, 23.4%), pleural fluid (PF, 4.5%), transbronchial needle aspiration samples (TBNA, 3.2%), pus (15.1%), liver abscess (0.5%), spinal aspirate (0.5%), endotracheal aspirate (ETA, 0.9%), gastric aspirate (GA, 5.5%), cold abscess (2.3%), and cerebrospinal fluid (CSF, 0.9%). Of these, 218 Xpert MTB/RIF Ultra positive specimens were enrolled in the study. Patients of all age groups and gender [female: 92 (42%) & male 126 (58%)] from out-patient department (OPD) and in-patients from various departments of AIIMS, New Delhi, India, were included in the study. (Fig. 1; Table 1).

### Diagnostic methods

All diagnostics methods were performed in accordance with the relevant microbiological guideline and regulations. Ziehl-Neelsen (ZN) staining was used to identify acid-fast bacilli (AFB) in the samples. Positive samples were recorded, and the results were compared accordingly (28). Xpert MTB/RIF Ultra assay used as a comparative method to evaluate MTB and RIF resistance status was as per the protocol previously published (13). Xpert MTB/XDR assay, performed on all Xpert MTB/RIF Ultra assay positive non-sputum specimens, was as per manufacturer’s instructions, to identify MTB and mutations associated with resistance to anti-TB drugs: INH, ETH, FLQ, AMK, KAN, and CAP (29). MGIT-960 liquid culture was deployed as a reference method for MTB detection. The cultures were monitored for growth, and positive cultures were subjected to further analysis for MTB identification (4). LPA (MTBDR*plus* and MTBDR*sl*) was performed on Xpert MTB/RIF Ultra Assay and MGIT culture positive specimens.

### Statistical analyses, observation, and interpretation

The diagnostic performance of the Xpert MTB/XDR assay was evaluated by comparing it to the Xpert MTB/RIF Ultra assay, MGIT-960 liquid culture & DST, ZN staining, and LPA (MTBDR*plus* & MTBDR*sl*). Key metrics analyzed included sensitivity, specificity, positive predictive value (PPV), and negative predictive value (NPV). Comparative analysis was conducted to evaluate the assay’s performance relative to other diagnostic methods using (Stata 12.1, Stata Corp., College Station, TX, USA) statistics software (30).

## Data Availability

The datasheets generated for this study are available in this article and also available from corresponding author on reasonable request.
